# Combined effect of dietary calcium consumption and physical activity on dental caries in children and adolescents: a study of the NHANES database

**DOI:** 10.1186/s12903-024-03969-5

**Published:** 2024-02-28

**Authors:** Qian Zhang, Xiaofan Bai, Huan Jin, Ning Dong

**Affiliations:** 1 Key laboratory of Shaanxi Province for Craniofacial Precision Medicine Research, College of Stomatology, Xi’an Jiaotong University, Xi’an, 710004 Shaanxi P.R. China; 2 Clinical Research Center of Shaanxi Province for Dental and Maxillofacial Diseases, College of Stomatology, Xi’an Jiaotong University, Zhongtu Building, No.85 North Street, Lianhu District, Xi’an, 710004 Shaanxi P.R. China; 3 Department of Pediatric Dentistry, College of Stomatology, Xi’an Jiaotong University, Xi’an, 710004 Shaanxi P.R. China

**Keywords:** Dietary ca intake, Physical activity, NHANES, Dental caries, Children and adolescents

## Abstract

**Background:**

Calcium (Ca) is a nutritional factor that associated with dental caries. A recent study showed that in the case of adequate Ca intake, a higher level of physical activity may contribute to bone mass accumulation. However, the combined effect between Ca intake and physical activity on caries experience is unclear. Herein, we aimed to explore the above combined effect on dental caries in children and adolescents.

**Methods:**

Data of 5,917 children and adolescents were extracted from the National Health and Nutrition Examination Surveys (NHANES) database in 2015–2020 in this cross-sectional study. The NHANES assessed the dietary Ca intake through the 24-hour dietary recalls, and the physical activity level was self-reported using the questionnaires. Also, the dental caries was diagnosed according to the Decayed, Missing and Filled Teeth/Surfaces (DMFT/S) index. Weighted univariate and multivariate logistic regression analyses were utilized to screen the covariates and to investigate the associations of dietary Ca intake and physical activity with dental caries, respectively, and assess the combined effect between dietary Ca intake and physical activity on dental caries. The evaluation indexes were odd ratios (ORs) and 95% confidence intervals (CIs). Subgroup analyses of age, obesity, and total sugar intake were also performed.

**Results:**

Among the eligible participants, 2,687 had caries experience. After adjusting for the covariates, we found that children and adolescents who not reach the recommendation level of Ca intake combined with physical activity less than 7 time in 1 week seemed to have higher odds of dental caries [OR = 1.77, 95%CI: (1.38–2.27)], compared with those who reached the standards. In addition, this potential combined effect was also found in age < 12 years old [OR = 1.62, 95%CI: (1.23–2.14)], non-obesity [OR = 1.88, 95%CI: (1.49–2.35)], and total sugar intake (all *P* < 0.05) subgroups.

**Conclusions:**

Ca intake and physical activity had a potential combined effect on dental caries in children and adolescents, but the causal relationships between them needed further clarification.

**Supplementary Information:**

The online version contains supplementary material available at 10.1186/s12903-024-03969-5.

## Background

Dental caries is one of the major chronic diseases threatening the oral health of children and adolescents, causing a considerable disease burden globally [1]. The etiology of dental caries is multifactorial, such as enamel hypoplasia, oral colonization with elevated levels of cariogenic bacteria, and the tooth-adherent bacteria metabolites [2]. Lacking of timely treatment, dental caries in children and adolescents can result in the development of an abscess or the death of a tooth [3]. Dental caries also affects general health in children and adolescents, and is associated with a number of diseases such as hypertension and type 1 diabetes [4, 5]. Therefore, it is essential to explore the risk factors in dental caries to improve and prevent dental caries in a young age.

The emphasis of dental caries prevention is shifting to the modifiable factors now, especially nutritional factors [6]. Higher calcium (Ca) and dairy intake may be associated with a lower risk of dental caries in children and adolescents. Madali et al. [7] indicated that dietary Ca intake was negatively associated with both the Decayed, Missing, Filled Teeth (DMFT) index and the Decayed, Missing and Filled Teeth/Surfaces (DMFT/S) index. Lempert et al. [8] investigated the association between the intake of dairy products and the development in dental caries among children/adolescents over a period of 3 and 6 years, and the results showed that dairy (including dairy Ca, whey and casein) and milk intake was generally inversely associated with caries experience (measured as DMFS). Lin et al. [9] also found daily intake of Ca was inversely associated with the caries index. High free-sugar intake is associated with dental caries, as well as increased weight and body mass index (BMI) [10]. Physical activity benefits to maintain normal BMI and health status, and however, the conclusions on the relationship between physical activity and dental caries are still ambiguous [11, 12]. A mutual etiologic contributing factor between obesity and dental caries is diet high in sugar, where physical activity can burn off the supernumerary energy (mostly roots in carbohydrates) and reduce BMI, and may also be associated with the development of dental caries [12]. It is worth noting that a systematic review has shown that Ca intake and physical activity may have a combined effect on bone mineral density (BMD) in children and adolescents, that is, in the case of adequate Ca intake, a higher level of physical activity may contribute to bone mass accumulation [13]. Herein, we speculated that adequate Ca intake combined with a high level of physical activity could benefit for the prevention of dental caries.

So far, no research has discussed whether there is a combined effect between Ca consumption and physical activity on dental caries in children and adolescents. Hence, investigating the combined effect of Ca with physical activity on development of dental caries may benefit to provide a multi-angle and comprehensive strategy for the prevention of dental caries clinically in children and adolescents, and improve the oral health condition more efficiently and comprehensively.

## Methods

### Study design and participants

Data of children and adolescents were extracted from the National Health and Nutrition Examination Surveys (NHANES) database in 2015–2020 in this cross-sectional study. The NHANES is jointly conducted by the Centers for Disease Control and Prevention (CDC) and the National Center for Health Statistics (NCHS) with the aim of assessing nutritional and health status of the noninstitutionalized children and adults in the United States. A complex, multistage stratified probability sampling method based on the selected counties, blocks, households, and persons within households were used in the NHANES. The NCHS trained professionals conducted interviews in participants’ homes, and the extensive physical examinations (including blood and urine collection) were conducted at mobile exam centers (MECs). For more details of the survey implementation please visit the website: https://www.cdc.gov/nchs/nhanes/index.htm.

Records of patient with caries experience were extracted from the database if they meet the inclusion criteria: (1) aged 2–17 years old (the age range commonly assessed in dental caries examinations) [14], (2) had the information on dietary Ca intake and physical activity, and (3) received the measurement for caries experience. A total of 5,917 children and adolescents were eligible. Since study data were de-identified and publicly available, and informed consent of all individuals have been obtained, no ethical approval from our institutional review board (IRB) was required. In addition, the current study adhered to the Declaration of Helsinki.

### Diagnosis of dental caries

The dental caries was diagnosed using the DMFT/S index according to the previous study [15], which is calculated by summing the combination of decayed (d or D), missing (M), and filled (f or F) teeth (t or T) or dental surfaces (s or S). The dmfts were used in primary teeth whereas the DMFTS were used in permanent teeth. The combinations of either dfs (decayed or filled surfaces in primary teeth) or DFS (decayed or filled surfaces in permanent teeth) were used for classification of dental caries presence. The score of DMFT/S index was ranged from 0 to 28, and in this study, we divided the children and adolescents into two groups: non-caries experience group (score = 0) and caries experience group (score > 0) [16].

### Assessment of dietary ca consumption

The measurement of dietary intake in the NAHENS was through two 24-hour dietary recalls [17]. The first 24-hour recall interview was conducted in person in the MEC by trained interviewers, and the second one was performed via telephone or mail 3–10 days later. The dietary questionnaires collected information on dietary intakes of Ca and its supplements. The dietary Ca intakes were divided into two levels according to the dietary reference intakes (DRIs): reach recommendation level and not reach recommendation level. Specific recommendation levels for dietary Ca intake of different age are shown in the Table S1.

### Evaluation of physical activity

The physical activity of children and adolescents was assessed by the NHANES questionnaires through the response to the question: “During the past week, on how many days did you exercise, play a sport, or participate in physical activity for at least 60 minutes?” Response options were: 0 days, 1–3 days, 4–6 days, or every day. We classified them into meeting physical activity guidelines group if they chose the response option of every day, otherwise into not meet physical activity guidelines group [18].

### Variables collection

We collected variables from the database including (1) demographic information: age, gender, race, poverty income ratio (PIR), household food security category, smoking during pregnancy, cotinine, (2) physical examination information: BMI, obesity, birth weight, (3) dietary intake information: total energy intake, total sugar intake, vitamin D (VD) intake, fiber intake, and (4) dental information: frequency of tooth brushing, amount of toothpaste used, and time period since last dental visit.

Obesity in children and adolescents was diagnosed according to the CDC standard, which was based on the BMI [19]. The BMI was calculated using the following formula: BMI = Weight (in kilograms)/Height (in meters)^2^. More details for CDC growth chat please visit: https://www.cdc.gov/growthcharts/html_charts/bmiagerev.htm. Blood samples of the participants were collected by the physical examination in MECs, and the serum cotinine was divided into three categories, including ≤ 0.05 ng/mL, > 0.05 ng/mL, and unknown. The dietary intakes of total energy, sugar, VD, and fiber were also collected using questionnaires of the NHANES 24-hour dietary recalls. Amount of toothpaste used was assessed according to the oral health questionnaire (OHQ-849), and was classified into two levels (< half load and ≥ half load). The frequency of tooth brushing was assessed through the question in OHQ-848G and OHQ-848Q: “How many times you brush your teeth in 1 day?”, and were divided into two levels including < 2 times in 1 day and ≥ 2 times in 1 day. In addition, the time period since last dental visit was also collected using the answers to the question OHQ-030 (which including < 1 year, 1–2 years, and > 2 years).

### Statistical analysis

Quantitative data were described using mean ± standard error (mean ± SE) and independent-samples t test was utilized for comparation between two groups. Enumeration data were expressed as number with constituent ratio [N (%)] and chi-square test (χ^2^) was employed for the comparison. We combined the data collected in 2015–2016 and 2017–2020 from the NHANES, which need a special weight for analyses. The following formulas were used to calculate the weights used in the database for different years to make them uniform: 2/5.2*WTDRD1 for data in 2015–2016 while 3.2/5.2*WTDRD1PP for data in 2017–2020. The weight “WTDRD1” was used because we included the dietary information from the first 24-hour dietary recall for analyses. Day 1 weights were constructed by taking the MEC sample weights (WTMEC2YR) and further adjusting for (a) the additional non-response and (b) the differential allocation by day of the week for the dietary intake data collection. More details about the NHANES special sample weights could be found elsewhere: https://wwwn.cdc.gov/Nchs/Nhanes/2015-2016/DR1TOT_I.htm#WTDRD1.

Weighted univariate logistic regression analysis was used to screen the covariates. Weighted univariate and multivariate logistic regression analyses were utilized to explore the association of dietary Ca intake and physical activity with dental caries, and assess the combined effect between dietary Ca intake and physical activity on dental caries. Also, we investigated these relationships in subgroups of age, BMI, and total sugar intake, because they are common influencing factors of dental caries among children and adolescents. The evaluation indexes were odds ratios (ORs) with 95% confidence intervals (CIs). Two-sided *P* < 0.05 was considered significant. Model 1 was the crude model. Model 2 adjusted for common demographic influencing factors, including age and race. Model 3 adjusted for all covariates that associated with dental caries (*P* < 0.05): age, race, PIR, household food security category, obesity, smoking during pregnant, cotinine, total energy intake, total sugar intake, amount of toothpaste use, and time period since last dental visit.

Statistical analysis was performed using SAS 9.4 (SAS Institute, Cary, NC, USA). Missing variables were shown in the Table S2. Missing data on the variable “cotinine” accounted for a large proportion so that we divided them into the “unknown” category. We used multiple imputation for the interpolation of other variables including missing data, and the sensitivity analysis of participants’ characteristics before and after the multiple imputation of missing data were shown in the Table S3.

## Results

### Characteristics of study population

**Fig. 1:**
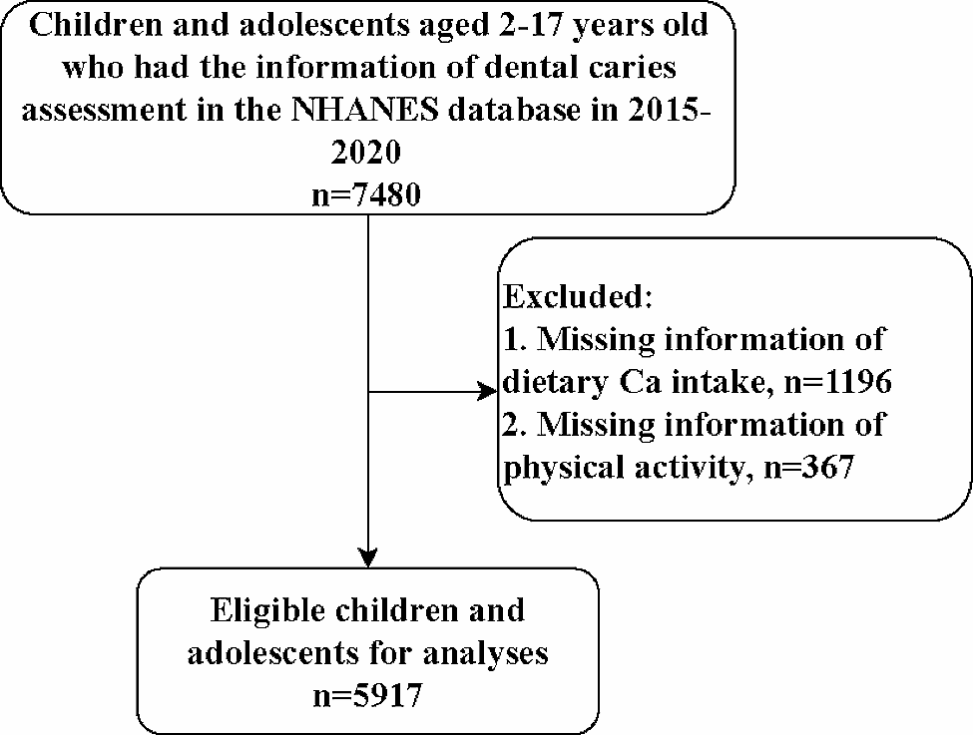
Flowchart of participants screening

Figure 1 shows the flowchat of participants screening. We initially included 7,480 children and adolescents aged 2–17 years old who had information on dental caries assessment from the database. Then those who without information on dietary Ca intake (*n* = 1196) or physical activity (*n* = 367) were excluded. Finally, 5,917 were eligible.

The characteristics of eligible children and adolescents are shown in the Table 1. A total of 2,687 children and adolescents had caries experience. The average age of participants was 9.29 years old, and 2,987 (50.92%) of them were male. The number of children and adolescents had physical activity of 7 times in 1 week between non-caries group and caries group was respectively 1,487 (45.46%) and 1,029 (34.52%). However, 3,975 (65.27%) of the participants had not reach the recommendation level of dietary Ca intake, whatever they had caries experience or not. In addition, race, PIR, household food security category, BMI, obesity, smoking during pregnancy, cotinine, total energy intake, total sugar intake, amount of toothpaste, time period since last dental visit, and the combined effect between dietary Ca intake and physical activity were all significantly different between the non-caries group and caries group (all *P* < 0.05).

**Table 1:** Characteristics of eligible children and adolescent

Variables	Total (*n* = 5917)	Non-caries (*n* = 3230)	Caries (*n* = 2687)	*P*
Age, years, Mean (S.E)	9.29 (0.10)	8.33 (0.17)	10.59 (0.18)	< 0.001
Age, n (%)				< 0.001
<12	4002 (64.66)	2336 (70.80)	1666 (56.39)	
≥12	1915 (35.34)	894 (29.20)	1021 (43.61)	
Gender, n (%)				0.263
Male	2987 (50.92)	1597 (49.86)	1390 (52.35)	
Female	2930 (49.08)	1633 (50.14)	1297 (47.65)	
Race, n (%)				< 0.001
Non-Hispanic White	1862 (51.34)	1136 (55.99)	726 (45.08)	
Non-Hispanic Black	1487 (13.58)	817 (13.74)	670 (13.36)	
Others	2568 (35.08)	1277 (30.28)	1291 (41.57)	
PIR, n (%)				< 0.001
<1.0	1780 (23.26)	829 (18.51)	951 (29.66)	
≥1.0	4137 (76.74)	2401 (81.49)	1736 (70.34)	
Household food security category, n (%)				< 0.001
Full food security	3251 (62.51)	1941 (68.25)	1310 (54.78)	
Marginal food security	964 (13.88)	491 (12.36)	473 (15.94)	
Low food security	1119 (15.68)	517 (12.71)	602 (19.68)	
Very low food security	583 (7.92)	281 (6.68)	302 (9.60)	
BMI, kg/m^2^, Mean (S.E)	19.96 (0.12)	19.28 (0.12)	20.87 (0.18)	< 0.001
Obesity, n (%)				< 0.001
No	4635 (79.39)	2598 (81.13)	2037 (77.05)	
Yes	1282 (20.61)	632 (18.87)	650 (22.95)	
Birth weight, pounds, n (%)				0.529
<5.5	912 (13.24)	491 (13.38)	421 (13.05)	
5.5-9.0	4574 (78.08)	2534 (78.59)	2040 (77.40)	
≥9.0	431 (8.68)	205 (8.03)	226 (9.55)	
Smoking during pregnant, n (%)				0.003
No	5164 (87.42)	2862 (89.36)	2302 (84.80)	
Yes	753 (12.58)	368 (10.64)	385 (15.20)	
Cotinine, ng/mL, n (%)				< 0.001
≤0.05	2472 (45.88)	1277 (43.44)	1195 (49.18)	
>0.05	1816 (27.12)	846 (23.65)	970 (31.79)	
Unknown	1629 (27.00)	1107 (32.91)	522 (19.03)	
Total energy intake, kcal, Mean (S.E)	1864.66 (14.37)	1807.87 (15.50)	1941.20 (28.15)	< 0.001
Total sugar intake, gm, Mean (S.E)	109.96 (1.08)	106.69 (1.42)	114.37 (1.87)	0.004
VD, gm, Mean (S.E)	7.85 (0.35)	8.23 (0.42)	7.33 (0.60)	0.230
Fiber intake, gm, Mean (S.E)	13.87 (0.18)	13.67 (0.24)	14.14 (0.21)	0.122
Frequency of tooth brushing, times/ 1 day, n (%)				0.836
<2	1980 (34.03)	1075 (33.78)	905 (34.36)	
≥2	3937 (65.97)	2155 (66.22)	1782 (65.64)	
Amount of toothpaste use, n (%)				< 0.001
<half load	2154 (39.42)	1285 (43.00)	869 (34.59)	
≥half load	3763 (60.58)	1945 (57.00)	1818 (65.41)	
Time period since last dental visit, years, n (%)				< 0.001
<1	4728 (80.43)	2436 (76.43)	2292 (85.82)	
1–2	458 (7.52)	233 (7.00)	225 (8.21)	
≥2	731 (12.05)	561 (16.56)	170 (5.97)	
Physical activity, n (%)				< 0.001
=7	2516 (40.80)	1487 (45.46)	1029 (34.52)	
<7	3401 (59.20)	1743 (54.54)	1658 (65.48)	
Ca intake, n (%)				< 0.001
Reach recommendation level	1942 (34.73)	1177 (38.19)	765 (30.06)	
Not reach recommendation level	3975 (65.27)	2053 (61.81)	1922 (69.94)	
Combined effect between Ca intake and physical activity, n (%)				< 0.001
Ca reach recommendation level & physical activity = 7	990 (16.70)	633 (19.68)	357 (12.67)	
Ca reach recommendation level & physical activity < 7	952 (18.03)	544 (18.51)	408 (17.39)	
Ca not reach recommendation level & physical activity = 7	1526 (24.11)	854 (25.78)	672 (21.85)	
Ca not reach recommendation level & physical activity < 7	2449 (41.16)	1199 (36.03)	1250 (48.09)	

Statistics analyses including χ^2^ test and t test

SE: standard error, PIR: poverty-to-income ratio, BMI: body mass index, VD: vitamin D, Ca: calcium

### Combined effect between dietary ca intake and physical activity on dental caries

We first screened the covariates associated with dental caries in children and adolescents (Table 2). The results showed that variables including age, race, household food security category, obesity, smoking during pregnancy, cotinine, total energy intake, total sugar intake, amount of toothpaste use, and time period since last dental visit were all significantly associated with dental caries (all *P* < 0.05).

**Table 2:** Univariate analysis to screen covariates associated with dental caries in children and adolescents

Variables	OR (95% CI)	*P*
Age		
<12	Ref	
≥12	1.87 (1.44–2.43)	< 0.001
Gender		
Male	Ref	
Female	0.91 (0.76–1.08)	0.270
Race		
Non-Hispanic White	Ref	
Non-Hispanic Black	1.21 (0.90–1.61)	0.197
Others	1.71 (1.38–2.11)	< 0.001
PIR		
<1.0	Ref	
≥1.0	0.54 (0.44–0.66)	< 0.001
Household food security category		
Full food security	Ref	
Marginal food security	1.61 (1.21–2.13)	0.002
Low food security	1.93 (1.48–2.52)	< 0.001
Very low food security	1.79 (1.31–2.44)	< 0.001
Obesity		
No	Ref	
Yes	1.28 (1.10–1.49)	0.002
Birth weight		
<5.5	Ref	
5.5-9.0	1.01 (0.83–1.22)	0.920
≥9.0	1.22 (0.76–1.96)	0.408
Smoking during pregnant		
No	Ref	
Yes	1.51 (1.15–1.98)	0.004
Cotinine		
≤0.05	Ref	
>0.05	1.19 (0.97–1.46)	0.097
Unknown	0.51 (0.40–0.65)	< 0.001
Total energy intake	1.01 (1.01–1.01)	< 0.001
Total sugar intake	1.01 (1.01–1.01)	0.002
VD	0.99 (0.98–1.01)	0.425
Fiber intake	1.01 (1.00-1.02)	0.133
Frequency of tooth brushing		
<2	Ref	
≥2	0.97 (0.76–1.25)	0.837
Amount of toothpaste use		
<half load	Ref	
≥half load	1.43 (1.22–1.67)	< 0.001
Time period since last dental visit		
<1	Ref	
1–2	1.04 (0.83–1.32)	0.715
≥2	0.32 (0.24–0.43)	< 0.001

PIR: poverty-to-income ratio, BMI: body mass index, VD: vitamin D

Then we explored the combined effect between dietary Ca intake and physical activity on dental caries in children and adolescents (Table 3). After adjusting for the above covariates, we found that children and adolescents who not reach recommendation level of dietary Ca intake [OR = 1.43, 95%CI: (1.22–1.67)] or having physical activity < 7 times in 1 week [OR = 1.27, 95%CI: (1.07–1.50)] had higher odds of dental caries. In addition, comparing to dietary Ca intake reached the recommendation level combined with physical activity at 7 times in 1 week, not reach the recommendation level of Ca intake combined with physical activity < 7 times in 1 week was associated with higher odds of dental caries [OR = 1.77, 95%CI: (1.38–2.27)].

**Table 3:** Combined effect between Ca intake and physical activity on dental caries

Variables	Model 1		Model 2		Model 3	
	OR (95% CI)	*P*	OR (95% CI)	*P*	OR (95% CI)	*P*
Ca intake						
Reach recommendation level	Ref		Ref		Ref	
Not reach recommendation level	1.44 (1.20–1.72)	< 0.001	1.28 (1.10–1.49)	0.002	1.43 (1.22–1.67)	< 0.001
Physical activity						
= 7	Ref		Ref		Ref	
< 7	1.58 (1.30–1.92)	< 0.001	1.26 (1.07–1.48)	0.007	1.27 (1.07–1.50)	0.007
Combined effect						
Ca reach recommendation level & physical activity = 7	Ref		Ref		Ref	
Ca reach recommendation level & physical activity < 7	1.46 (1.07–1.99)	0.018	1.26 (0.94–1.68)	0.123	1.22 (0.91–1.62)	0.173
Ca not reach recommendation level & physical activity = 7	1.32 (1.01–1.72)	0.046	1.28 (0.99–1.66)	0.062	1.38 (1.08–1.76)	0.012
Ca not reach recommendation level & physical activity < 7	2.07 (1.54–2.80)	< 0.001	1.59 (1.23–2.06)	< 0.001	1.77 (1.38–2.27)	< 0.001
*P* for trend	< 0.001		< 0.001		< 0.001	

Ca: calcium, OR: odds ratio, CI: confidence interval, Ref: reference

Model 1: crude model;

Model 2: adjusted for age and race;

Model 3: adjusted for age, race, PIR, household food security category, obesity, smoking during pregnant, cotinine, total energy intake, total sugar intake, amount of toothpaste use, and time period since last dental visit

### The combined effect between Ca intake and physical activity on dental caries in age, obesity, and total sugar intake subgroups

We further explored this combined effect in children and adolescents with different age, obesity status, and total sugar intake levels (Table 4). Similarly, we found the relationship ofnot reach recommendation level of Ca intake combined with physical activity < 7 times in 1 week with high odds of caries in children and adolescents who aged < 12 years old [OR = 1.62, 95%CI: (1.23–2.14)] or not have obesity [OR = 1.88, 95%CI: (1.49–2.35)]. Besides, whether the total sugar intake > 98 gm, children and adolescents who had not reach the recommendation level of dietary Ca intake combined with physical activity < 7 times in 1 week had high odds of caries experience (≤ 98 gm: OR = 2.71 and > 98 gm: OR = 1.39).

**Table 4:** Combined effect between Ca intake and physical activity on dental caries in age, obesity, and total sugar intake subgroups

Subgroups	OR (95% CI)	*P*	OR (95% CI)	*P*
**Age**	**< 12 years old (** ***n*** ** = 4002)**		**≥ 12 years old (** ***n*** ** = 1915)**	
Ca reach recommendation level & physical activity = 7	Ref		Ref	
Ca reach recommendation level & physical activity < 7	1.24 (0.91–1.70)	0.165	1.04 (0.52–2.07)	0.910
Ca not reach recommendation level & physical activity = 7	1.45 (1.12–1.88)	0.006	0.94 (0.40–2.18)	0.880
Ca not reach recommendation level & physical activity < 7	1.62 (1.23–2.14)	0.001	1.56 (0.65–3.73)	0.312
*P* for trend	0.001		0.068	
**Obesity**	**No (** *n* ** = 4635)**		**Yes (** *n* ** = 1282)**	
Ca reach recommendation level & physical activity = 7	Ref		Ref	
Ca reach recommendation level & physical activity < 7	1.26 (0.98–1.62)	0.069	0.97 (0.44–2.12)	0.930
Ca not reach recommendation level & physical activity = 7	1.47 (1.14–1.89)	0.004	0.93 (0.55–1.57)	0.789
Ca not reach recommendation level & physical activity < 7	1.88 (1.49–2.35)	< 0.001	1.28 (0.60–2.75)	0.511
*P* for trend	< 0.001		0.248	
**Total sugar intake**	**≤ 98 gm (** *n* ** = 3010)**		**> 98 gm (** *n* ** = 2907)**	
Ca reach recommendation level & physical activity = 7	Ref		Ref	
Ca reach recommendation level & physical activity < 7	2.02 (1.24–3.30)	0.006	0.94 (0.66–1.36)	0.751
Ca not reach recommendation level & physical activity = 7	2.06 (1.36–3.14)	0.001	1.11 (0.80–1.55)	0.524
Ca not reach recommendation level & physical activity < 7	2.71 (1.70–4.32)	< 0.001	1.39 (1.02–1.89)	0.038
*P* for trend	< 0.001		0.006	

Ca: calcium, OR: odds ratio, CI: confidence interval, Ref: reference

Age subgroups: adjusted for race, PIR, household food security category, obesity, smoking during pregnant, cotinine, total energy intake, total sugar intake, amount of toothpaste use, and time period since last dental visit;

Obesity subgroups: adjusted for age, race, PIR, household food security category, smoking during pregnant, cotinine, total energy intake, total sugar intake, amount of toothpaste use, and time period since last dental visit;

Total sugar intake subgroups: adjusted for age, race, PIR, household food security category, obesity, smoking during pregnant, cotinine, total energy intake, amount of toothpaste use, and time period since last dental visit

## Discussion

We explored the roles of dietary Ca intake and physical activity in dental caries among children and adolescents in this cross-sectional study. Our findings indicated that dietary Ca consumption not reach the recommended level or physical activity less than 7 times in a week were both associated with higher odds of dental caries. A potential combined effect between dietary Ca intake and physical activity was found linked to the odds of caries. Also, this combined effect was observed in subgroups of < 12 years old, non-obesity, and all levels of total sugar intake.

To the best of our knowledge, it was the first time to investigate the combined effect between dietary Ca intake and physical activity on childhood caries experience. Julián-Almárcegui et al. [13] conducted a systematic review on the combined effects of physical activity and diet on bone mass accrual in children and adolescents, and the results of randomized controlled trials showed that physical activity has a potential effect on improving bone health under conditions of adequate dietary Ca intake. Ca is one of the key macroelements make up the bulk of the mineralized human tissues. Therefore, adequate consumption of Ca is of crucial importance in maintaining the health, function and retention of teeth and bones [20]. Singal et al. [21] concluded that topical treatment using calcium phosphate and fluoride (CaP + F) group showed good remineralization potential and the antibacterial effect on dental caries among children. In agreement with the current study, Pratyusha et al. [22] conducted a comparative cross-sectional study on association of serum VD and salivary Ca level in 3-11-year-old schoolchildren with dental caries, indicating that VD deficiency and lower salivary Ca levels may be the potential risk factors for the occurrence of dental caries. The composition of salivary microbial community was highly correlated with Ca, and the cariogenic microorganisms present in the oral cavity play a key role in the occurrence and development of childhood caries, and the formed biofilm can metabolize carbohydrates to produce organic acids, resulting in local pH decline and demineralization of dental hard tissues [23]. Differently, in this study, we assessed the dietary Ca intake level, which may have specific underlying mechanisms to explain the relationship of deficient dietary Ca intake and high odds of dental caries. In addition, the average VD consumption in participants was 7.85 gm that with no significant difference between caries group and non-caries group. Nevertheless, Sadashivappa et al. [24] considered there is no statistically significant association of dental caries with salivary Ca level. Besides, few studies have explored the relationship between physical activity and caries in children and adolescents, and they reached inconsistent conclusions. Sanchez et al. [25] assessed the relationship between physical activity level and oral health in Spanish adults, and concluded that physical activity is favorably associated with some of the self-reported oral health correlates. However, Chauhan et al. [26] showed that caries activity increased immediately after a vigorous workout and remains high at least for 15 min among adults belonged to 4 races. Due to the limitation of different measurement standards and populations, the relationship between physical activity and caries in children and adolescents needed further interpretation.

The decreased level of Ca results in a reduction of enamel crystallinity, increasing its retentive surface, and further decreasing overall resistance [24]. A study conducted to compare the salivary Ca levels between patients with caries affected versus 32 non-caries individuals and found that Ca levels in non-caries group were far higher than caries group [22]. Another study in a sample of Canadian and American preschool-aged children showed that higher VD and Ca concentrations were significantly and independently associated with lower odds of severe early childhood caries [27]. Both higher VD and Ca concentrations were associated with lower odds for enamel hypoplasia, and these associations may be plausible biologically. The disturbances of VD and Ca during the development of tooth may result in dentin and enamel defects, which can further increase the risk of caries [27]. Previous studies have shown that it is likely to be driven by physical activity promoting a positive inflammatory profile, which subsequently lowers the risk of oral diseases [26]. The potential mechanisms of the potential benefits of physical activity on caries may be participation in physical activity lower the levels of inflammatory markers, such as C-reactive protein, tumor necrosis factor alpha, and interleukin 6, which are instrumental in the pathogenesis of oral disorders [28, 29]. Besides, physical activity has also been shown to increase the antimicrobial proteins and lactoferrin concentration, and lead to a decrease in inflammation [30]. However, the specific underlying mechanism of physical activity on childhood caries is still unclear, which needed further basic studies to clarify. We recommended that adequate intake of dietary Ca in children and adolescents, which is conducive to the development of dental structure and function; at the same time, combined with appropriate physical exercise, excess energy (mainly from carbohydrates) can be consumed, which is conducive to alleviating oxidative stress, reducing inflammatory response, regulating the diversity of caries causing microorganisms, and thus playing a potential role in preventing dental caries. Herein, we considered the research results could provide some references for public health policies and preventative strategies in clinical practice.

The subgroup analysis showed the similar results that the combined effect between dietary Ca intake and physical activity on caries was also found in children and adolescents who aged < 12 years old or not have obesity. In addition, whether the participants had a total sugar intake over 98 gm or not, failing to reach the recommendation of Ca intake and the physical activity standard was a potential risk factor for caries. Similar to the study conducted by Pratyusha et al. [22], we found this combined effect significantly in age < 12 years old subgroup. However, most of the participants in the current study was less than 12 years old (64.66%) and Non-Hispanic White, so that the results may influenced by the study samples. Also, the influencing factors for the age difference of caries in children and adolescent are diverse. Previous studies have reported that prolonged breastfeeding [31] or the epigenetic change such as DNA methylation [32] may be associated with the caries in the early age. Obesity and dental caries share some common and modifiable factors, such as diet and lifestyle [33]. A systematic review and meta-analyses by Manohar et al. [34] showed that children with overweight and obesity had a significantly higher dental caries experience compared with those who with normal weight. Similarly, our subgroup analysis results showed that among children and adolescents without obesity, dietary Ca intake not reach recommendation level combined with physical activity less than 7 times a week was associated with higher odds of caries than those who reached the standards. However, Shi et al. [33] considered that obesity is negatively associated with dental caries in children and adolescents in Huizhou, China. The possible reason for these different results may be that the target population is different. In addition, those obese children and adolescents may come from economically wealthy families and their parents believe that higher food intake makes their children grow faster, which results in overweight or obesity, and they also pay more attention to their child’s health status, including oral health. It is well-known the role of sugars in the etiology of dental caries, where the metabolism of sugars by dental biofilm results in dysbiosis, pH drops, and consequently tooth demineralization [35]. Sugar results in biofilm accumulation and dysbiosis locally [36], and is involved in oxidative stress and low-grade systemic inflammation systemically [37]. Sufficient Ca consumption and physical activity may benefit to reduce the oxidative stress as well as improving inflammatory state that further related to decreased risk of dental caries. Besides, sugar and sweetened beverages intake is associated with both increased acid production by cariogenic plaque bacteria and energy consumption in children [38]. Commonly, children and adolescents with Ca deficiency and have deficient physical activity may meanwhile consume more sugar. However, in the current research, no matter how much of total sugar intake (more than 98 gm or ≤ 98 gm), relationship of combined effect between dietary Ca intake and physical activity on dental caries was significant, indicating that both high and low sugar consumption individuals need to focus the dietary Ca intake as well as appropriate daily physical activity. The median total sugar intake per day in children and adolescents in the NHANES was 98 gm, which is similar to the previous NHANES report (about 100 gm) [39]. Due to this consumption difference in this population, specific mechanisms to explain the observed combined effect on dental caries need further exploration.

This study explored the combined effect between dietary Ca consumption and physical activity on risk of dental caries, and we hope the results can provide some references for the exploration for lifestyle intervention methods to prevent and control dental caries in children and adolescents. Basing on the NHANES database, which uses multi-stage complex sampling, the study sample size is large and has a good representation of the United States populations. However, there are still some limitations in the current study. Because of the cross-sectional nature of this study, we are unable to clarify the causal association of Ca intake and physical activity with dental caries in children and adolescents. However, we tried our best to adjust for the covariates as much as possible to reduce the bias caused by confounding factors. Data on dietary intake information were collected through the NHANES 24-hour dietary recalls with questionnaires, which is hard to avoid the recall bias, but we only included the first day’s records to minimize the recall bias. Additionally, due to the information on specific treatment of dental caries, such as dental sealants and root canal therapy, was not available in the database, we could not extracted and included in the analyses.

## Conclusion

Dietary Ca intake combined with physical activity have a potential negative association with caries experience in children and adolescents. Further studies are needed to explore the causal relationship between this effect and dental caries.

### Electronic supplementary material

Below is the link to the electronic supplementary material.

Supplementary Material 1

## Data Availability

The datasets used and/or analyzed during the current study are available from the NHANES database, https://wwwn.cdc.gov/nchs/nhanes/.

## Abbreviations

Ca: Calcium
DMFT: Decayed, Missing, Filled Teeth
DMFT/S: Decayed, Missing and Filled Teeth/Surfaces
BMI: Body mass index
BMD: Bone mineral density
NHANES: National Health and Nutrition Examination Surveys
CDC: Centers for Disease Control and Prevention
NCHS: National Center for Health Statistics
MECs: Mobile exam centers
DRIs: Dietary reference intakes
PIR: Poverty income ratio
VD: Vitamin D
mean ± SE: Mean ± standard error
ORs: Odds ratios
CIs: Confidence intervals

## References

[1] Wen PYF, Chen MX, Zhong YJ, Dong QQ, Wong HM (2022). Global Burden and Inequality of Dental Caries, 1990 to 2019. J Dent Res.

[2] Zou J, Du Q, Ge L, Wang J, Wang X, Li Y (2022). Expert consensus on early childhood caries management. Int J Oral Sci.

[3] Matthews P (2023). Prevention of dental caries in children and young people. Nurs Child Young People.

[4] Ostalska-Nowicka D, Paszyńska E, Dmitrzak-Węglarz M, Neyman-Bartkowiak A, Rabiega A, Zachwieja J (2021). Dental caries-related primary hypertension in children and adolescents: cross-sectional study. Oral Dis.

[5] Gunasekaran S, Silva M, O’Connell MA, Manton DJ, Hallett KB (2022). Caries experience and gingival health in children and adolescents with type 1 diabetes mellitus-A cross-sectional study. Pediatr Diabetes.

[6] Shivakumar S, Srivastava A (2018). Body Mass Index and Dental caries: a systematic review. Int J Clin Pediatr Dent.

[7] Madali B, Inan-Eroglu E, Ozsin-Ozler C, Karahan S, Uzamis-Tekcicek M, Buyuktuncer Z (2023). Development and validation of a comprehensive food frequency questionnaire that assesses the dietary intake related with dental health in children: a pilot study. Clin Nutr ESPEN.

[8] Lempert SM, Christensen LB, Froberg K, Raymond K, Heitmann BL (2015). Association between dairy intake and caries among children and adolescents. Results from the Danish EYHS follow-up study. Caries Res.

[9] Lin HS, Lin JR, Hu SW, Kuo HC, Yang YH (2014). Association of dietary calcium, phosphorus, and magnesium intake with caries status among schoolchildren. Kaohsiung J Med Sci.

[10] Kopycka-Kedzierawski DT, Auinger P, Billings RJ, Weitzman M (2008). Caries status and overweight in 2- to 18-year-old US children: findings from national surveys. Community Dent Oral Epidemiol.

[11] Iglehart JK (1994). Health policy report. Health care reform. The States. N Engl J Med.

[12] Alam BF, Abbasi N, Hussain T, Khan MA, Chaudhary MAG, Ijaz F (2022). Relationship of BMI with the diet, physical activity and oral hygiene practices amongst the dental students. BMC Oral Health.

[13] Julian-Almarcegui C, Gomez-Cabello A, Huybrechts I, Gonzalez-Aguero A, Kaufman JM, Casajus JA (2015). Combined effects of interaction between physical activity and nutrition on bone health in children and adolescents: a systematic review. Nutr Rev.

[14] Sanders AE, Slade GD (2018). Blood lead levels and Dental Caries in U.S. children who do not drink tap Water. Am J Prev Med.

[15] Dye BA, Mitnik GL, Iafolla TJ, Vargas CM. Trends in dental caries in children and adolescents according to poverty status in the United States from 1999 through 2004 and from 2011 through 2014. J Am Dent Assoc. 2017;148:550–65. e7. 10.1016/j.adaj.2017.04.013 28619207

[16] Herzog K, Scott JM, Hujoel P, Seminario AL (2016). Association of vitamin D and dental caries in children: findings from the National Health and Nutrition Examination Survey, 2005–2006. J Am Dent Assoc.

[17] Moshfegh AJ, Rhodes DG, Baer DJ, Murayi T, Clemens JC, Rumpler WV (2008). The US Department of Agriculture Automated Multiple-Pass Method reduces bias in the collection of energy intakes. Am J Clin Nutr.

[18] Friel CP, Duran AT, Shechter A, Diaz KM (2020). U.S. children meeting physical activity, screen time, and Sleep guidelines. Am J Prev Med.

[19] Ogden CL, Kuczmarski RJ, Flegal KM, Mei Z, Guo S, Wei R (2002). Centers for Disease Control and Prevention 2000 growth charts for the United States: improvements to the 1977 National Center for Health statistics version. Pediatrics.

[20] Lippert F (2020). Chapter 3: macroelements: ca, na, K. P Cl Monogr Oral Sci.

[21] Singal K, Sharda S, Gupta A, Malik VS, Singh M, Chauhan A (2022). Effectiveness-of calcium phosphate derivative agents on the prevention and remineralization of caries among children- A systematic review & meta-analysis of randomized controlled trials. J Evid Based Dent Pract.

[22] Pratyusha N, Vinay C, Uloopi KS, RojaRamya KS, Ahalya P, Devi C (2021). Association of serum Vitamin D and salivary calcium and phosphorus levels in 3-11-year-old schoolchildren with dental caries. J Indian Soc Pedod Prev Dent.

[23] Lin X, Wang Y, Ma Z, Xie M, Liu Z, Cheng J (2023). Correlation between caries activity and salivary microbiota in preschool children. Front Cell Infect Microbiol.

[24] Sadashivappa Pateel DG, Gunjal S, Dutta S (2022). Association of Salivary Statherin, Calcium, and Proline-Rich proteins: a potential predictive marker of Dental Caries. Contemp Clin Dent.

[25] Sanchez GFL, Smith L, Koyanagi A, Grabovac I, Yang L, Veronese N (2020). Associations between self-reported physical activity and oral health: a cross-sectional analysis in 17,777 Spanish adults. Br Dent J.

[26] Chauhan A, Mazlee AM, Azhar NA, Ng Bansing SA, Qing CS, Sidhu DS (2020). Effect of HIIT (high-intensity interval training) on vulnerability to dental caries. J Oral Biol Craniofac Res.

[27] Williams TL, Boyle J, Mittermuller BA, Carrico C, Schroth RJ. Association between Vitamin D and Dental Caries in a sample of Canadian and American preschool-aged children. Nutrients. 2021;13. 10.3390/nu13124465 PMC8706858 34960016

[28] Zheng G, Qiu P, Xia R, Lin H, Ye B, Tao J (2019). Effect of Aerobic Exercise on inflammatory markers in healthy middle-aged and older adults: a systematic review and Meta-analysis of Randomized controlled trials. Front Aging Neurosci.

[29] Conrads G, About I (2018). Pathophysiology of Dental Caries. Monogr Oral Sci.

[30] Gillum T, Kuennen M, McKenna Z, Castillo M, Jordan-Patterson A, Bohnert C (2017). Exercise increases lactoferrin, but decreases lysozyme in salivary granulocytes. Eur J Appl Physiol.

[31] van Meijeren-van Lunteren AW, Voortman T, Elfrink MEC, Wolvius EB, Kragt L (2021). Breastfeeding and Childhood Dental Caries: results from a socially diverse birth Cohort Study. Caries Res.

[32] Mj S, Jm NM, Dj C, Mc MRS (2022). DNA methylation in childhood dental caries and hypomineralization. J Dent.

[33] Shi R, Lin C, Li S, Deng L, Lin Z, Xiu L (2022). Obesity is negatively associated with dental caries among children and adolescents in Huizhou: a cross-sectional study. BMC Oral Health.

[34] Manohar N, Hayen A, Fahey P, Arora A (2020). Obesity and dental caries in early childhood: a systematic review and meta-analyses. Obes Rev.

[35] Nyvad B, Takahashi N (2020). Integrated hypothesis of dental caries and periodontal diseases. J Oral Microbiol.

[36] Costa SA, Nascimento GG, Colins PMG, Alves CMC, Thomaz E, Carvalho Souza SF (2022). Investigating oral and systemic pathways between unhealthy and healthy dietary patterns to periodontitis in adolescents: a population-based study. J Clin Periodontol.

[37] Lula EC, Ribeiro CC, Hugo FN, Alves CM, Silva AA (2014). Added sugars and periodontal disease in young adults: an analysis of NHANES III data. Am J Clin Nutr.

[38] Palacios C, Joshipura K, Willett W (2009). Nutrition and health: guidelines for dental practitioners. Oral Dis.

[39] Marriott BP, Hunt KJ, Malek AM, Newman JC (2019). Trends in Intake of Energy and Total Sugar from Sugar-Sweetened beverages in the United States among children and adults, NHANES 2003–2016. Nutrients.

