# Supplementary material for: Combined effect of dietary calcium consumption and physical activity on dental caries in children and adolescents: a study of the NHANES database
Source: BMC Oral Health. 2024 Feb 28;24:281. doi: 10.1186/s12903-024-03969-5 (PMC10900671; doi:10.1186/s12903-024-03969-5)
Supplement: Supplementary file 1 — Supplementary Material 1 [file 12903_2024_3969_MOESM1_ESM.docx]

**Table S1 Recommendation of dietary Ca intake in children and adolescents with different age and gender**

| Age (years) | Ca (mg/day) |
| --- | --- |
| 1-3 | 700 |
| 4-8 | 1,000 |
| **Males** | |
| 9-13 | 1,300 |
| 14-17 | 1,300 |
| **Females** | |
| 9-13 | 1,300 |
| 14-17 | 1,300 |

Ca: calcium.

**Table S2 Missing data and the proportions**

| Variables | n (%) |
| --- | --- |
| PIR | 513 (8.67%) |
| Household food security category | 203 (3.43%) |
| Cotinine | 1629 (27.53%) |
| BMI | 90 (1.52%) |
| Birth weight | 651 (11%) |
| Time period since last dental visit | 15 (0.25%) |
| Smoking during pregnant | 533 (9.01%) |
| Frequency of tooth brushing | 515 (8.7%) |
| Amount of toothpaste use | 562 (9.5%) |

PIR: poverty-to-income ratio, BMI: body mass index

**Table S3 Sensitivity analysis of patients’ characteristics before and after the multiple imputation of missing data**

| Variables | Before the imputation | After the imputation | Statistics | *P* |
| --- | --- | --- | --- | --- |
| PIR, n (%) |  |  | χ^2^=3.44 | 0.064 |
| <1.0 | 1601 (22.79) | 1780 (23.26) |  |  |
| ≥1.0 | 3803 (77.21) | 4137 (76.74) |  |  |
| Household food security category, n (%) |  |  | χ^2^=2.83 | 0.419 |
| Full food security | 3144 (62.69) | 3251 (62.51) |  |  |
| Marginal food security | 926 (13.84) | 964 (13.88) |  |  |
| Low food security | 1083 (15.62) | 1119 (15.68) |  |  |
| Very low food security | 561 (7.85) | 583 (7.92) |  |  |
| BMI, kg/m^2^, Mean (S.E) | 19.97 (0.13) | 19.96 (0.12) | t=0.83 | 0.410 |
| Birth weight, pounds, n (%) |  |  | χ^2^=2.47 | 0.292 |
| <5.5 | 817 (13.50) | 912 (13.24) |  |  |
| 5.5-9.0 | 4062 (78.19) | 4574 (78.08) |  |  |
| ≥9.0 | 387 (8.30) | 431 (8.68) |  |  |
| Smoking during pregnant, n (%) |  |  | χ^2^=2.30 | 0.130 |
| No | 4720 (87.86) | 5164 (87.42) |  |  |
| Yes | 664 (12.14) | 753 (12.58) |  |  |
| Frequency of tooth brushing, times/1 day n (%) |  |  | χ^2^=1.21 | 0.272 |
| <2 | 1777 (33.74) | 1980 (34.03) |  |  |
| ≥2 | 3625 (66.26) | 3937 (65.97) |  |  |
| Amount of toothpaste use, n (%) |  |  | χ^2^=0.51 | 0.474 |
| < half load | 1964 (39.61) | 2154 (39.42) |  |  |
| ≥ half load | 3391 (60.39) | 3763 (60.58) |  |  |
| Time period since last dental visit, years, n (%) |  |  | χ^2^=3.80 | 0.150 |
| <1 | 4718 (80.46) | 4728 (80.43) |  |  |
| 1-2 | 457 (7.52) | 458 (7.52) |  |  |
| ≥2 | 727 (12.02) | 731 (12.05) |  |  |

χ^2^: chi-square test, t: t test.

PIR: poverty-to-income ratio, BMI: body mass index
